# Effectiveness of interventions to improve adherence to antidepressant medication in patients with depressive disorders: a cluster randomized controlled trial

**DOI:** 10.3389/fpubh.2024.1320159

**Published:** 2024-04-02

**Authors:** Tasmania del Pino-Sedeño, Himar González-Pacheco, Beatriz González de León, Pedro Serrano-Pérez, Francisco Javier Acosta Artiles, Cristina Valcarcel-Nazco, Isabel Hurtado-Navarro, Cristobalina Rodríguez Álvarez, María M. Trujillo-Martín, on behalf of MAPDep Team

**Affiliations:** ^1^Canary Islands Health Research Institute Foundation (FIISC), Tenerife, Spain; ^2^Evaluation Unit (SESCS), Canary Islands Health Service (SCS), Tenerife, Spain; ^3^Network for Research on Chronicity, Primary Care, and Health Promotion (RICAPPS), Barcelona, Spain; ^4^Faculty of Health Sciences, Universidad Europea de Canarias, Tenerife, Spain; ^5^Multiprofessional Teaching Unit of Family and Community Care La Laguna-Tenerife Norte, Management of Primary Care of Tenerife, Santa Cruz de Tenerife, Spain; ^6^Department of Psychiatry, Hospital Álvaro Cunqueiro, SERGAS, Vigo, Spain; ^7^Department of Psychiatry and Forensic Medicine, Autonomous University of Barcelona, Barcelona, Spain; ^8^Translational Neuroscience Research Group, Galicia Sur Health Research Institute (IIS-Galicia Sur), SERGAS-UVIGO, CIBERSAM, Vigo, Spain; ^9^Service of Mental Health, General Management of Healthcare Programs, The Canary Islands Health Service, Las Palmas, Gran Canaria, Spain; ^10^Department of Psychiatry, University Hospital of Gran Canaria Doctor Negrín, Las Palmas, The Canary Islands, Spain; ^11^Health Services Research Unit, Foundation for the Promotion of Health and Biomedical Research of Valencia Region (FISABIO), Valencia, Spain; ^12^Department of Preventive Medicine and Public Health, University of La Laguna, Tenerife, Canary Islands, Spain; ^13^Institute of Biomedical Technologies (ITB), University of La Laguna, Tenerife, Canary Islands, Spain

**Keywords:** depression, treatment adherence and compliance, antidepressive agents, Spain, randomized controlled trial

## Abstract

**Aim:**

To assess the effectiveness of two interventions of knowledge transfer and behavior modification to improve medication adherence in patients with depressive disorders.

**Methods:**

An open, multicenter, three-arm clinical trial with random allocation by cluster to usual care or to one of the two interventions. The intervention for psychiatrists (PsI) included an educational program based on a patient-centered care model. The intervention for patients and relatives (PtI) included a collaborative care program plus a reminder system that works using an already available medication reminder application. The primary outcome was patient adherence to antidepressant treatment assessed through the Sidorkiewicz Adherence Instrument. Secondary measures were depression severity, comorbid anxiety and health-related quality of life. Mixed regression models with repeated measures were used for data analysis.

**Results:**

Ten psychiatrists and 150 patients diagnosed with depressive disorder from eight Community Mental Health Units in the Canary Islands (Spain) were included. Compared with usual care, no differences in long-term adherence were observed in either group PsI or PtI. The PsI group had significantly improved depression symptoms (*B* = −0.39; 95%CI: −0.65, −0.12; *p* = 0.004) during the follow-up period. The PtI group presented improved depression symptoms (*B* = −0.63; 95%CI: −0.96, −0.30; *p* < 0.001) and mental quality of life (*B* = 0.08; 95%CI: 0.004, 0.15; *p* = 0.039) during the follow-up period.

**Conclusion:**

The assessed interventions to improve adherence in patients with depressive disorder were effective for depression symptoms and mental quality of life, even over the long term. However, no effect on antidepressant adherence was observed.

## 1 Introduction

Depression is a common mental health issue that affects people all over the world and is one of the main reasons for disability globally (1). The COVID-19 pandemic has exacerbated this situation (2), with an estimated 53 million additional cases of major depressive disorder worldwide. Currently, it is estimated that 264 million people suffer from depression (3).

The consequences of these disorders in terms of health loss are enormous. Depressive disorders are currently the leading contributor to global disability, accounting for over 1.8% of all Disability-Adjusted Life Years (DALYs) in 2019 (1). Additionally, depression is linked to a reduction in the average life expectancy by 15 years (4) and is considered the primary contributing factor to suicide, resulting in 700,000 deaths per year (5).

The economic impact of depression is profound and extends to individuals, families, and society. According to the World Health Organization (WHO), depression is estimated to cost over $1 trillion per year globally (6). This cost is expected to increase in the coming years as the prevalence of depression continues to rise. Besides, depression disorders not only have cost effects, but these conditions can also reduce productivity (6), increase social isolation (7), social stigma (8), and drug abuse (9).

Despite effective treatments being available (10), compliance has a high dropout rate and adherence to antidepressant medication remains a challenge for healthcare providers (11–13). In Spain, studies have found non-adherence rates ranging from 20 to 70% (14–16). Non-adherence compromises the effectiveness of available treatments, interferes with patient recovery and represents a significant burden for healthcare systems (17). Conversely, proper adherence increases the likelihood of preventing relapse and recurrence, reducing healthcare costs, improving depression symptoms, lowering rates of emergency visits and hospitalizations, enhancing work productivity and reducing healthcare costs (17–19), thus benefiting the patient, healthcare systems and society at large (20).

Pharmacological adherence is influenced by several factors, including the patient’s clinical and sociodemographic characteristics, the disease itself, medication, healthcare professionals, family and friends, media and society (21–23). To address adherence issues effectively, multiple approaches consider these factors with the aim of improving medication adherence and treatment outcomes. A recently published systematic review has shown that multicomponent interventions improve short and medium-term adherence to medication among adults with depressive disorders, but there is insufficient evidence in the literature to draw longer-term conclusions (24). However, the evidence strongly supports the effectiveness of collaborative care in improving adherence among adults with depressive disorders (24). These results corroborated the notion that collaborative care has the potential to be not only clinically beneficial for addressing symptom management in adults with depressive disorders (25, 26), but also to significantly enhance treatment adherence. Therefore, it can be assumed that a multifaceted intervention targeting all dimensions affecting medication adherence problems, i.e., the patient, the healthcare provider and the healthcare delivery system, could improve medication adherence in these cases more than single-component strategies.

There is a need for evidence-based effective strategies involving patients and physicians that improve short- and long-term adherence to depression treatment. The Adherence Improvement in Patients with Depression study (*Mejora de la Adherencia en Pacientes con Depresión*, MAPDep) was designed with this in mind. *Our hypothesis was that strategies involving patients and physicians would increase antidepressant pharmacological therapeutic adherence and improve the patients’ health outcomes throughout the 12-month follow-up*. The aim of MAPDep is to assess the effectiveness of these strategies to improve medication adherence in patients with depressive disorders in a period of 12 months after the intervention.

## 2 Materials and methods

### 2.1 Trial design

An open, multicenter, three-arm clinical trial with random allocation by cluster to usual care or one of two interventions of knowledge transfer and behavior modification was conducted (trial registration number: ClinicalTrials.gov NCT03668457). One intervention was aimed at psychiatrists (PsI), and other intervention was aimed at patients and family members (PtI). In the control group, patients received the usual care provided by the Canary Islands Health Service (SCS). The full study protocol has been published before (27).

When the recruitment of professionals and patients was about to begin, the COVID-19 pandemic was declared, significantly hindering the ability to maintain a normal recruitment pace. As a result, before randomization, a decision was made to eliminate one of the initially planned arms, which combined intervention both to patients and professionals.

### 2.2 Participants

#### 2.2.1 Selection criteria

##### 2.2.1.1 Community mental health units

Urban or rural Community Mental Health Units (CMHU) within the SCS were selected.

##### 2.2.1.2 Mental health professionals

Psychiatrists who carry out their care activity in a CMHU with stability in the position during the development of the study were included.

##### 2.2.1.3 Patients

Adults 18 years and over with a diagnosis of depressive disorder (ICD10: F32-F33, Episodes and recurrent depressive disorder), with antidepressant drug treatment, who could read and understand Spanish, with sufficient cognitive abilities to answer the questionnaires and regularly used a mobile smartphone were included. Subsequently, those who agreed to participate were sent the study information sheet and signed the informed consent form. Those who had not attended a medical appointment in the previous six months, or who were participating in another experimental research study, or with a history of severe psychiatric illness (ICD-10: F30-31, Manic episode and bipolar disorder and ICD-10: F20-29, Schizophrenia, schizotypal disorder and delusional disorders), or with a physical disability that limits participation in group activities, or in remission phase, or pregnancy were excluded.

#### 2.2.2 Setting and recruitment

This study was performed in CMHUs in Tenerife, La Gomera, El Hierro and Gran Canaria, (Canary Islands, Spain). All CMHUs were invited to participate through an information meeting with their coordinators on each island.

The selection of participants was carried out in two stages. In the first one, psychiatrists working in CMHUs were contacted by e-mail or telephone to explain the objectives of the study and to request their collaboration in March 2020. Before or during medical consultations, the psychiatrists who agreed to collaborate consecutively invited patients who met the selection criteria for the study and sought their initial agreement to participate. In a subsequent phone call, researchers expanded the information about the project and confirmed their participation. Patients were recruited between April 2021 and November 2021. Accepting patients signed an informed consent form and completed the baseline questionnaires on their first visit.

The 12-month follow-up timeline started directly after the conclusion of each professional’s intervention and the completion of each patient’s intervention.

### 2.3 Intervention

This consisted of interventions that were articulated toward two agents: the patients or the psychiatrists.

#### 2.3.1 Intervention for mental health professionals

The PsI aims to update clinical knowledge in patient-centered care (PCC), including shared decision-making (SDM) and motivational interviewing methods, based on the available evidence, and to train the clinician in the necessary skills to improve the drug adherence of patients with depressive disorders.

A series of 4-h group sessions consisting of two interactive online sessions, one month apart, led by a clinical leader along with the principal investigator were set up. The contents and procedures of the sessions were designed based on the results of the literature review carried out in a previous study (24), selecting the best documented and evaluated interventions providing valid data on the results of adherence to pharmacological treatment and over the long term.

The content of the first session was intended to foster skills in promoting SDM within the framework of the PCC model. This session used a series of short video films featuring role-playing exercises involving diverse scenarios with complex sham patients.

In the second session, the content was aimed at promoting motivational interviewing methods, as well as effective communication and negotiation abilities.

The sessions were led by mental health professionals with expertise in PCC methods and communication skills.

#### 2.3.2 Intervention to patients with depressive disorders

The design of the PtI was based on the Chronic Care Model, incorporating components of depression education, medication management, behavioral activation, and the use of a high-quality medication reminder mobile app already available on the market (24, 28). Its aim was not only to increase adherence to pharmacological treatment but also to improve the training and strengthening of patients in self-motivation, self-control and monitoring of their disease.

There were individual telephone sessions led by two general health psychologists. Patients were scheduled to receive a series of 6–12 contacts with them over a period not exceeding 14 weeks. The frequency of contacts was adapted to the individual needs of each patient. However, weekly contacts were recommended during the initial 4–5 weeks, followed by bi-weekly contacts thereafter. If the patient and investigator mutually agreed, more frequent sessions could be organized. In most cases, the maximum number of sessions was limited to ten.

The initial session was planned to take place face-to-face. The pandemic situation led to considering its replacement by telephone contact. The first session lasted approximately 30–40 min. Subsequent sessions were planned and conducted via the phone. The timing and day of each telephone contact were negotiated with the patient. These sessions lasted between 15 and 20 min, with the possibility of shorter sessions.

The design of the contents and procedures of the sessions were based on the results of the literature review, selecting, as in the case of professionals, the best documented and evaluated interventions providing valid and long-term data on the improvement of drug adherence. This intervention was comprised of three components: depression education, medication management and behavioral activation.

Prior to these sessions, patients were instructed in the use of a reminder system using a mobile application (APP) over the 12-month follow-up. The instruction consisted of a video tutorial and a posterior phone call to clarify any doubts. This instruction was given by a family doctor familiar with the use of the APP. The APP was selected according to the results of the APP evaluation conducted in the Spanish market (28).

#### 2.3.3 Control

Patients included in the control group received the usual care provided by the SCS, including antidepressant therapy and referral for other treatments. Psychiatrists did not have access to PtI.

### 2.4 Outcomes

#### 2.4.1 Primary outcome

Patient adherence to antidepressant drug treatment was assessed through the Sidorkiewicz Adherence Instrument validated in Spanish (29). The instrument consists of five items, with three having multiple answers and two with dichotomous answers. This instrument showed adequate predictive validity (29). Results range from 1 (high adherence) to 6 (drug suspension). For some analyses, the original adherence level for each drug was grouped into two categories: ‘adherent’ (including values 1, 2, and 3, representing high, good, and moderate adherence, respectively) versus ‘non-adherent’ (including values 4, 5, and 6, representing poor, very poor, and discontinuation, respectively). Patient classification as adherent or non-adherent was based on their adherence to all medications within their antidepressant treatment regimen. Adherence was measured at baseline, 3 months, 6 months and 12 months.

#### 2.4.2 Secondary outcomes

Emotional distress was assessed using the Spanish version of Hospital Anxiety and Depression Scale (HADS) (30); severity of depressive symptoms using the Spanish version of Beck Depression Inventory-II (BDI-II) (31); and self-reported multidimensional health-related quality of life (HRQoL) evaluated with the 12-item Short Form Survey (SF-12) (32, 33). Secondary outcomes were assessed at baseline, 3 months, 6 months, and 12 months.

HADS is a valid and reliable 14-item self-reporting scale, containing two seven-item scales, one for anxiety and one for depression. Each item is rated on a four-point Likert scale (0–3), and the scores for both scales range from 0 to 21. Higher scores indicate greater impairment (30).

BDI-II is a reliable and well-validated 21-item self-report multiple-choice inventory designed to measure the severity of depression. Each item is rated on a four-point scale (0–3), and the total score ranges from 0 to 63. The severity score is classified as follows: 0–13 (minimal depression), 14–19 (mild depression), 20–28 (moderate depression), and 29–63 (severe depression). The minimal clinically important difference was defined as a 17.5% reduction in scores from baseline (34).

The 12-item Short Form Survey (SF-12) assesses multidimensional HRQoL, including physical and mental health domains. It uses categorical (e.g., yes/no) and Likert response formats to evaluate various aspects of well-being (physical functioning, role physical, bodily pain, general health, vitality, social functioning, role emotional and mental health). Higher scores on the SF-12 indicate better HRQoL. The SF-12 is a reliable and well-validated instrument (35, 36).

#### 2.4.3 Additional outcomes

In addition, the following measures were collected at baseline from the patients: patient attitudes toward psychopharmacological medication were assessed using the Spanish version of the Drug Attitude Inventory-10 Items (DAI-10) (37); patient beliefs about treatment were measured with the Beliefs about Medicines Questionnaire (BMQ) (38); patients’ belief in their ability to control health was assessed with the Multidimensional Health Locus of Control (MHLC) scale (39); the role of patients in decision-making was determined using the Control Preferences Scale (CPS) (40); and reactance proneness was measured using the Hong Psychological Reactance Scale (HPRS) (41), which assesses the motivational state resulting from perceived freedom elimination or threat, potentially impacting the effectiveness of persuasive messages. Sociodemographic and clinical data were also collected from both the patients and the psychiatrists.

The data was obtained from two different sources: the patients themselves, and the professionals. Questionnaires on baseline sociodemographic and clinical characteristics (depressive disorder and duration of the disorder), DAI-10, BMQ, MHLC; CPS and HPRS; the Sidorkiewicz Adherence Instrument, the HADS, the BDI- II and the SF-12 scale. A research assistant, who was a general health psychologist, was recruited for data collection and entry. In the case of the professionals, a questionnaire of basic characteristics (baseline) was applied. In patients, all measurements were collected by telephone. The information from all professionals was collected via email prior to the initial group session in the arm where the professionals underwent intervention. All the information was stored in a protected EXCEL document that met the required confidentiality criteria.

### 2.5 Sample size calculation

A two-tailed test with a 5% level of significance, a power of 80% and an intra-cluster correlation coefficient of 0.01, considering a 10% loss to follow-up, 100 patients and two professionals per intervention arm were required to detect a minimum difference of 30% in treatment adherence between the intervention and control groups, measured by the Sidorkiewicz Adherence Instrument.

However, as previously mentioned, the COVID-19 situation slowed down the recruitment process, and a total sample of only 150 patients was achieved.

### 2.6 Randomization

Randomization was conducted at the CMHU level to usual care or one of two interventions (PsI or PtI). There was no stratification at the professional or patient level. Allocation was performed by clusters using an automatic generation of random number to assign CMHUs to their respective groups. After the recruitment of professionals and the selection of patients who were consecutively recruited by their psychiatrist were completed, including the clinical leader, the principal investigator, mental health professionals, and general health psychologists, was ready to implement the interventions, was ready to implement the interventions, the study statistician sent the allocation of the CMHU directly to the principal investigator.

### 2.7 Blinding

Participating psychiatrists and patients from each selected CMHU were not assigned to intervention (groups 1–3) until the last patient was recruited. Psychiatrists and patients could not be blinded after assignment to interventions or control group due to the nature of the interventions.

The investigator responsible for executing the final analyses was blinded to the intervention assignment.

### 2.8 Statistical analyses

Continuous variables were summarized using means and standard deviations (SD), while categorical variables were presented as counts and percentages. Baseline characteristics of patients were compared using one-way analysis of variance (ANOVA) for continuous variables, and for categorical variables, either the Pearson chi-square test or Fisher’s exact test was used.

The investigators conducted multivariate regressions to explore the factors associated with multiple outcome measures, including adherence, depression, anxiety, and HRQoL. The analysis included independent factors such as patient characteristics (both sociodemographic and clinical) and potential predictor variables related to personality, cognitive and behavioral aspects (DAI-10, BMQ, MHLC, CPS and HPRS).

Although multilevel mixed models were planned to incorporate cluster effects across three levels (patients, psychiatrists, and CMHU), due to the limited sample size, only the patient level was included in the analyses. Mixed-effects regression models with repeated measures were applied, adjusting for the interaction between time and the intervention group, as well as their main effects. Covariates were included if their bivariate contrast with the dependent variable yielded a *p*-value <0.10. A linear link function was used for continuous dependent variables, and a logistic function was used for dichotomous dependent variables.

All the analyses were performed on an intention-to-treat basis. Multiple imputation by chained equations (MICE) was used for missing values (See Supplementary Material). Statistical analyzes were performed using STATA version 17.0 (42).

## 3 Results

**Figure 1:**
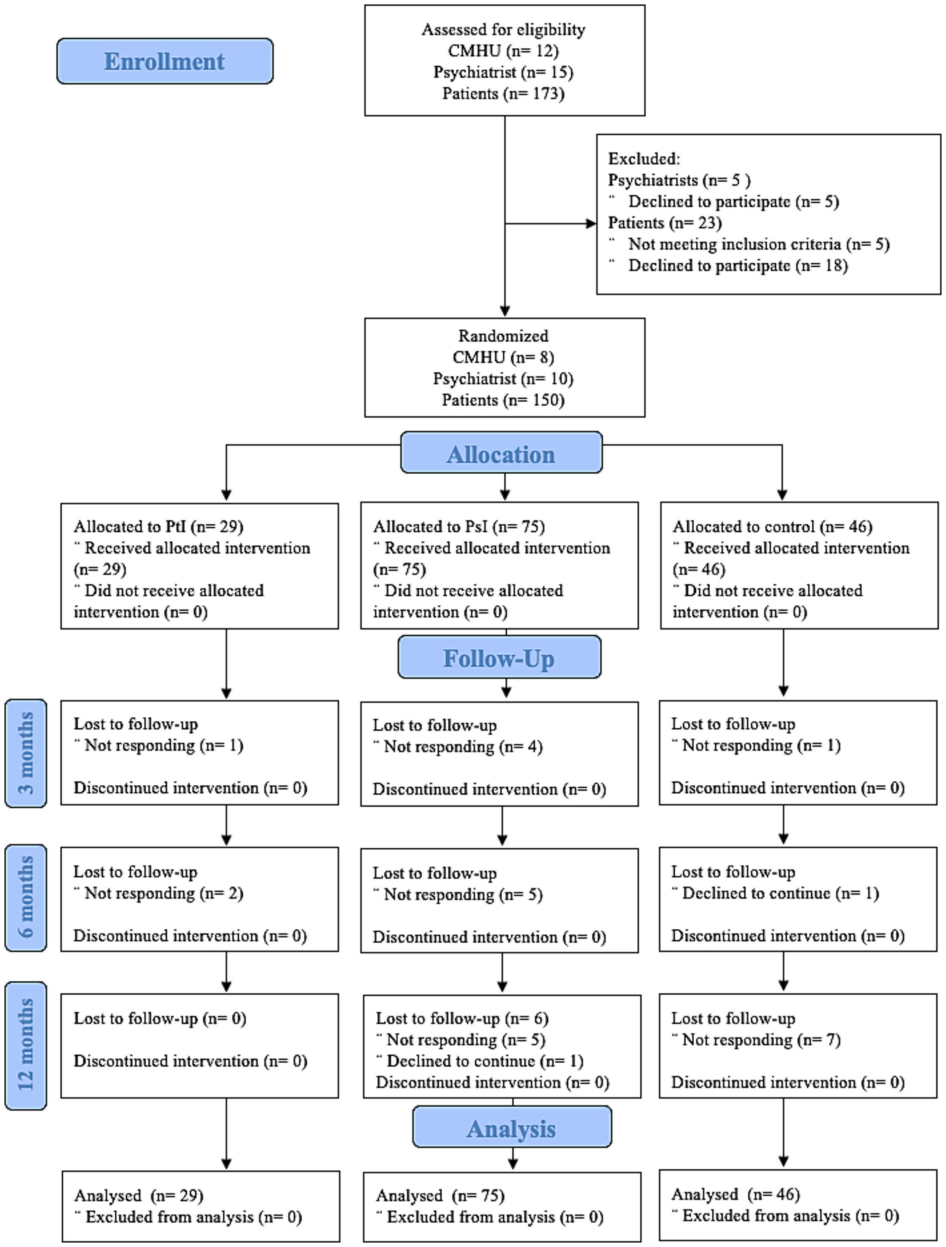
Consolidated Standards of Reporting Trials (CONSORT) flow diagram. CMHU, Community Mental Health Units; PsI, intervention for patients; PtI, intervention for psychiatrists.

Ten psychiatrists agreed to participate in the study voluntarily. Out of the 173 patients who were identified by psychiatrists, 150 individuals were included in the study and randomized to receive the intervention for patients with depressive disorder (*n* = 29), intervention for psychiatrists (*n* = 75) or usual care (*n* = 46) (see Figure 1).

### 3.1 Sample characteristics

Fifty percent of the psychiatrists who agreed to participate in the study were women, 60% worked at CMHU in Gran Canaria, with an average of 15.9 years (SD 11.7) of experience (see Table 1).

**Table 1:** Baseline sociodemographic and clinical characteristics of the psychiatrists.

		*N* = 10
Age, mean (SD)		45.6 (11.3)
Age, range		29-66
*Gender, n (%)*		
	Women	5 (50.0)
	Men	5 (50.0)
*Island, n (%)*		
	Gran Canaria	6 (60.0)
	Tenerife	2 (20.0)
	El Hierro	1 (10.0)
	La Gomera	1 (10.0)
Years of professional experience, mean (SD)		15.9 (11.7)
Years of professional experience, range		2-37

SD, Standard deviation.

Most of the patients were women (73.3%), with an average age of 57.28 years (SD 9.88) and were diagnosed with a depressive episode (79.4%). On average, participants had had their diagnosis for 7.66 years (SD 5.87) and presented a severe level of depression (mean 29.91; SD 12.64). Roughly half of the individuals (47.3%) were either married or in a committed relationship, while 50% had limited education, either having no formal schooling or only having completed primary studies (see Table 2).

**Table 2:** Baseline sociodemographic and clinical characteristics of the patients.

Characteristics		Total (*N* = 150)	Intervention to psychiatrists (*n* = 75)	Intervention to patients (*n* = 29)	Control (*n* = 46)	*p*-value
Age, mean (SD)		57.28 (9.88)	58.03 (10.05)	51.96 (10.25)	59.42 (8.28)	**0.004**
Age, range		29-80	29-80	30-69	37-76	
*Gender, n (%)*						
	Women	110 (73.3)	55 (73.3)	21 (72.4)	34 (73.9)	0.990
	Men	40 (26.7)	20 (26.7)	8 (27.6)	12 (26.1)	
*Educational level, n (%)*						
	No formal education or primary studies	75 (50)	39 (52)	12 (41.4)	24 (52.2)	0.586
	Secondary or higher studies	75 (50)	36 (48)	17 (58.6)	22 (47.8)	
*Marital status, n (%)*						
	In a relationship	71 (47.3)	34 (45.3)	12 (41.4)	25 (54.3)	0.487
	Single/widowed/separated	79 (52.7)	41 (54.7)	17 (58.6)	21 (45.7)	
*Cohabitation status, n (%)*						
	Live alone	32 (21.3)	16 (21.3)	7 (24.1)	9 (19.6)	0.895
	Do not live alone	118 (78.7)	59 (78.7)	22 (75.9)	37 (80.4)	
*Parental status, n (%)*						
	Yes	129 (86)	67 (89.3)	25 (86.2)	37 (80.4)	0.366
	No	21 (14)	8 (10.7)	4 (13.8)	9 (19.6)	
*Type of diagnosis* * *, n (%)*						
	F32-F33 Depressive episodes and recurrent depressive disorder	108 (79.4)	51 (78.5)	25 (86.2)	32 (76.2)	0.571
	F34 Dysthymic disorder	28 (20.6)	14 (21.5)	4 (13.8)	10 (23.8)	
Time since diagnosis, mean (SD)		7.66 (5.87)	8.12 (5.64)	4.98 (5.85)	8.81 (5.80)	**0.017**
Total antidepressants, mean (SD)		1.48 (0.72)	1.57 (0.64)	1.28 (0.65)	1.46 (0.81)	0.148
Total drugs, mean (SD)		2.95 (1.22)	2.87 (1.18)	3.21 (1.21)	2.91 (1.31)	0.438
Drug, range		1-7	1-7	1-5	1-6	
*Polypharmacy, n (%)*						
	Yes	133 (88.7)	67 (89.3)	27 (93.1)	39 (84.8)	0.583
	No	17 (11.3)	8 (10.7)	2 (6.9)	7 (15.2)	
*Antidepressant treatment adherence (Sidorkiewicz), n (%)*						
	No	32 (21.3)	14 (18.7)	7 (24.1)	11 (23.9)	0.728
	Yes	118 (78.7)	61 (81.3)	22 (75.9)	35 (76.1)	
BDI-II, mean (DS)		29.91 (12.64)	26.52 (12.93)	33.90 (10.85)	32.93 (11.92)	**0.004**
*HADS, mean (SD)*						
	Anxiety	17.51 (3.89)	18 (3.90)	15.79 (3.71)	17.78 (3.77)	**0.028**
	Depression	16.65 (2.14)	16.57 (1.98)	16.72 (2.31)	16.72 (2.33)	0.917
SF-12, mean (SD)						
	Physical health	13.11 (1.93)	13.05 (1.76)	13.41 (2.15)	13.02 (2.07)	0.647
	Mental health	16.31 (2.34)	16.25 (2.33)	16.14 (2.36)	16.52 (2.38)	0.751
*BMQ, mean (SD)*						
	General Harm	12.62 (3.01)	12.53 (2.95)	12.10 (2.98)	13.11 (3.12)	0.347
	General Overuse	9.59 (2.44)	9.67 (2.24)	8.86 (2.59)	9.91 (2.63)	0.179
	Specific Necessity	18.15 (3.68)	17.89 (3.85)	18.45 (3.74)	18.37 (3.40)	0.701
	Specific Concern	14.39 (3.91)	14.09 (3.59)	14.79 (4.38)	14.61 (4.16)	0.647
*CPS, n (%)*						
	Collaborative	57 (38.0)	25 (33.3)	13 (44.8)	19 (41.3)	0.477
	Passive	93 (62.0)	50 (66.7)	16 (55.2)	27 (58.7)	
*MHLC scale, mean (SD)*						
	Internal locus of control	19.86 (3.19)	19.88 (3.16)	20.97 (2.92)	19.13 (3.25)	0.051
	External locus of control, chance	16.37 (3.10)	16.48 (2.64)	15.83 (3.86)	16.52 (3.29)	0.582
	External locus of control, doctor	12.00 (1.76)	12.09 (1.74)	11.76 (1.70)	12 (1.85)	0.688
	External locus of control, other people	10.87 (2.01)	11.15 (1.62)	10.34 (2.73)	10.76 (2.04)	0.171
*HPRS, mean (SD)*						
	Total	36.41 (10.37)	34.40 (10.46)	39.52 (10.0)	37.72 (9.98)	**0.045**
	Affective	18.91 (5.61)	18.07 (5.59)	21.07 (5.72)	18.91 (5.33)	**0.049**
	Cognitive	17.50 (5.74)	16.33 (5.79)	18.45 (5.17)	18.80 (5.74)	**0.043**

* *N* = 136; BDI-II, Beck Depression Inventory-II; BMQ, Beliefs about Medicines Questionnaire; CPS, Control Preferences Scale; HADS, Hospital Anxiety and Depression Scale; HPRS, Hong Psychological Reactance Scale; MHLC, Multidimensional Health Locus of Control; SD, Standard deviation; SF-12, Short Form-12.

Patients were prescribed 2.95 antidepressant medications on average (range: 1–7). Initially, 78.7% of patients adhered to the prescribed antidepressant treatment.

For a comprehensive overview of the sample characteristics, including sociodemographic and clinical factors considered in the study, as well as the descriptive data for primary and secondary variables over time, please see Tables 2, 3, respectively.

**Table 3:** Descriptive data for primary and secondary variables over time.

	Baseline	M3	M6	M12
*Antidepressant treatment adherence (Yes) (Sidorkiewicz), %*				
Intervention to patients (*n* = 29)	75.9	71.73	72.14	71.07
Intervention to psychiatrists (*n* = 75)	81.3	81.69	70.28	76.90
Control (*n* = 46)	76.1	57.78	70.89	69.33
*BDI-II, mean (SD)*				
Intervention to patients (*n* = 29)	33.90 (10.85)	32.89 (11.45)	28.12 (10.11)	29.99 (11.85)
Intervention to psychiatrists (*n* = 75)	26.52 (12.93)	25.46 (12.75)	27.63 (14.34)	24.87 (13.22)
Control (n = 46)	32.93 (11.92)	30.74 (12.57)	35.51 (13.19)	35.33 (11.61)
*HADS – Anxiety, mean (SD)*				
Intervention to patients (*n* = 29)	15.79 (3.71)	16 (3.30)	16.67 (2.88)	16.82 (3.19)
Intervention to psychiatrists (*n* = 75)	18 (3.90)	17.73 (3.73)	18.12 (3.94)	18.48 (3.45)
Control (n = 46)	17.78 (3.77)	18.08 (3.64)	16.90 (4.05)	17.00 (4.01)
*HADS – Depression, mean (SD)*				
Intervention to patients (*n* = 29)	16.72 (2.31)	16.04 (2.20)	16.33 (1.76)	17.12 (1.98)
Intervention to psychiatrists (*n* = 75)	16.57 (1.98)	16.54 (2.10)	16.33 (2.10)	16.21 (1.92)
Control (*n* = 46)	16.72 (2.33)	16.92 (2.39)	16.76 (2.42)	16.64 (2.09)
*SF-12 – Physical QoL, mean (SD)*				
Intervention to patients (*n* = 29)	13.41 (2.15)	13.54 (1.88)	13.41 (2.24)	13.40 (1.90)
Intervention to psychiatrists (*n* = 75)	13.05 (1.76)	13.1 (2.05)	12.85 (1.74)	12.98 (1.57)
Control (*n* = 46)	13.02 (2.07)	12.98 (1.95)	13.18 (1.66)	13.25 (1.69)
*SF-12 – Mental QoL, mean (SD)*				
Intervention to patients (*n* = 29)	16.14 (2.36)	16.04 (2.43)	15.89 (1.87)	16.35 (1.48)
Intervention to psychiatrists (*n* = 75)	16.25 (2.33)	16.43 (2.12)	16.37 (2.12)	16.17 (1.69)
Control (*n* = 46)	16.52 (2.38)	16.03 (2.25)	15.78 (2.06)	15.74 (1.69)

BDI-II, Beck Depression Inventory-II; HADS, Hospital Anxiety and Depression Scale; M3, 3 months; M6, 6 months; M12, 12 months; QoL, quality of life; SD, Standard deviation; SF-12, Short Form-12.

### 3.2 Impact of the intervention for patients and for psychiatrists on adherence

No difference was found over time on patients´ adherence to antidepressant treatment. No difference was observed between the PsI, PtI, and the control groups over the follow-up period (see Table 4).

**Table 4:** Multivariate mixed regression model for intervention sample.

	Adherence		
	OR	95%CI	*p*-value
Time	1.01	0.90, 1.13	0.886
*Intervention (ref: Control)*			
Patients	0.36	0.07, 2.0	0.244
Psychiatrists	0.43	0.12, 1.59	0.205
Time* Intervention (ref: Control)			
Patients	1.02	0.85, 1.23	0.800
Psychiatrists	1.06	0.92, 1.22	0.437
Time since diagnosis	0.96	0.86, 1.06	0.390
BMQ *–* Specific Necessity	1.18	1.02, 1.37	**0.027**
*CPS: Passive* (ref: collaborative)	0.23	0.08, 0.68	**0.008**
MHLC scale *–* External locus of control, other people	0.80	0.61, 1.04	0.100
Intercept	0.43	0.03, 12.0	0.619

Non-adherence.

BMQ, Beliefs about Medicines Questionnaire; CPS, Control Preferences Scale; MHLC, Multidimensional Health Locus of Control; OR: Odds Ratio. Bold values are statistically significant.

However, the more passive in decision-making patients had greater adherence than the collaborative patients (odds ratio, OR = 0.23; 95%CI: 0.08, 0.68; *p* = 0.008). Besides, a high BMQ score for the sub-scale of perception of need for medication was associated with less antidepressant adherence (OR = 1.18; 95%CI: 1.02, 1.37; *p* = 0.027) (see Table 4).

### 3.3 Impact of the interventions on depression severity

In the case of the control group, the follow-up time showed a significant association with depression severity (*B* = 0.29; 95%CI: 0.09, 0.49; *p* = 0.005), suggesting a gradual increase in depression levels over time in this group (see Table 5).

**Table 5:** Multivariate mixed regression model for intervention sample.

	BDI-II		
	*B*	95%CI	*p*-value
Time	0.29	0.09, 0.49	**0.005**
*Intervention* (ref: Control)			
Patients	1.32	−3.42, 6.07	0.615
Psychiatrists	−3.88	−7.56, −0.19	**0.039**
Time*Intervention (ref: Control)			
Patients	−0.63	−0.96, −0.30	**<0.001**
Psychiatrists	−0.39	−0.65, −0.12	**0.004**
BMQ *–* Specific Necessity	1.46	1.03, 1.88	**<0.001**
MHLC scale *–* Internal locus of control	−0.64	−1.13, −0.15	**0.010**
HPRS *–* Affective	0.42	0.14, 0.71	**0.003**
Intercept	9.59	−3.96, 23.13	0.165

Depression.

BDI-II, Beck Depression Inventory-II; BMQ, Beliefs about Medicines Questionnaire; HPRS, Hong Psychological Reactance Scale; MHLC, Multidimensional Health Locus of Control. Bold values are statistically significant.

Regarding the intervention effect, the PtI group exhibited a significant reduction in depression levels over time compared to the control group (*B* = –0.63; 95%CI: –0.96, –0.30; *p* = <0.001), while the PsI group also showed a less pronounced decrease compared to the control group (*B* = –0.39; 95%CI: –0.65, –0.12; *p* = 0.004). However, right from the baseline, the PsI group exhibited a markedly lower level of depression compared to the control group (*B* = –3.88; 95%CI: –7.56, –0.19; *p* = 0.039) and neither intervention group achieved a minimal clinically important decrease (see Table 5).

Additionally, a higher BMQ score for the subscale of perception of need for medication (*B* = 1.46; 95%CI: 1.03, 1.88; *p* < 0.001), a higher score in the subscale that measures an affective dimension of reactance of MHLC (*B* = –0.64; 95%CI: –1.03, –1.88; *p* = 0.01), and a lower internal locus of control score (*B* = 0.42; 95%CI: 0.14, 0.71; *p* = 0.003) were associated with higher severity of depression (see Table 5).

### 3.4 Impact of the interventions on emotional distress

The follow-up time showed a significant association with anxiety levels in the control group (*B* = –0.09; 95%CI: –0.14, –0.04; *p* = 0.001), suggesting a slight decrease in anxiety levels over time in this group (see Table 6).

**Table 6:** Multivariate mixed regression model for intervention sample.

	HADS – Anxiety			HADS – Depression		
	B	95%CI	*p*-value	B	95%CI	*p*-value
Time	−0.09	−0.14, −0.04	**0.001**	−0.02	−0.06, 0.03	0.489
*Intervention* (ref: Control)						
Patients	−2.01	−3.57, −0.44	**0.012**	−0.19	−1.06, 0.69	0.678
Psychiatrists	−0.13	−1.36, 1.09	0.832	−0.11	−0.79, 0.58	0.762
Time* Intervention (ref: Control)						
Patients	0.17	0.08, 0.25	**<0.001**	0.07	−0.01, 0.14	0.076
Psychiatrists	0.14	0.07, 0.21	**<0.001**	−0.02	−0.08, 0.04	0.482
*Gender: Women* (ref: Men)	–	–	–	−0.65	−1.29, −0.02	**0.043**
*Cohabitation status: Live alone* (ref: Do not live alone)	–	–	–	0.72	0.04, 1.40	**0.038**
MHLC scale – Internal locus of control	–	–	–	−0.19	−0.28, −0.11	**<0.001**
MHLC scale – External locus of control, chance	−0.33	−0.50, −0.17	**<0.001**	−0.01	−0.10, 0.08	0.786
HPRS – Affective	−0.14	−0.23, −0.05	**0.003**	–	–	–
Intercept	26.03	22.63, 29.44	<0.001	21.04	18.73, 23.35	<0.001

Emotional distress.

HADS, Hospital Anxiety and Depression Scale; HPRS, Hong Psychological Reactance Scale; MHLC, Multidimensional Health Locus of Control. Bold values are statistically significant.

Concerning the intervention effect, the PtI (*B* = 0.17; 95%CI: 0.08, 0.25; *p* **<** 0.001) and PsI (*B* = 0.14; 95%CI: 0.07, 0.21; *p* **<** 0.001) intervention groups showed a progressive increase in anxiety levels over time compared to the control group. However, starting from the baseline, the PtI group demonstrated a lower anxiety level compared to the control group (*B* = –2.01; 95%CI: –3.57, –0.44; *p* = 0.012; see Table 6).

Additionally, an increase in MHLC for the subscale external locus of control, chance (patients’ belief that one’s health condition is a matter of fate, luck or chance) (*B* = –0.33; 95%CI: –0.50, –0.17; *p* < 0.001) or in the subscale that measures an affective dimension of reactance of MHLC (*B* = –0.14; 95%CI: –0.23, –0.05; *p* = 0.003) were associated with a decrease in anxiety levels (see Table 6).

No significant differences were found over time and/or between the interventions and control groups in patients’ depression levels assessed with HADS (see Table 6). However, women presented lower depression levels than men, as indicated by a negative coefficient (*B* = –0.65; 95%CI: –1.29, –0.02; *p* = 0.043). On the other hand, patients living alone showed higher levels of depression than those who live accompanied (*B* = 0.72; 95%CI: 0.04, 1.40; *p* = 0.038). Once again, a lower internal locus of control score (*B* = –0.19; 95%CI: –0.28, –0.11; *p* < 0.003) was associated with higher severity of depression (see Table 6).

### 3.5 Impact of the interventions on physical and mental quality of life

No significant difference was found in physical QoL over time and/or between the interventions and control groups. Only an increase in age was associated with a decrease in physical QoL.

The mental QoL in the control group showed a gradual decrease over time (*B* = –0.06; 95%CI: –0.10, –0.01; *p* = 0.012). Regarding the intervention effect over time, there was an impact on mental QoL. Patients who received the PtI demonstrated a slight increase in mental QoL over time compared to the control group (*B* = 0.08; 95%CI: 0.004, 0.15; *p* = 0.039). Although the PsI group also exhibited a similar trend, it did not reach statistical significance (*B* = 0.05; 95%CI: –0.003, 0.11; *p* = 0.062). Finally, single, widowed or separated patients showed lower levels of mental QoL than those who were in a relationship (*B* = –0.70; 95%CI: –1.29, –0.11; *p* = 0.020) (see Table 7).

**Table 7:** Multivariate mixed regression model for intervention sample.

	SF-12 – Physical QoL			SF-12 – Mental QoL		
	*B*	95%CI	*p*-value	*B*	95%CI	*p*-value
*Time*	0.02	−0.01, 0.05	0.229	−0.06	−0.10, −0.01	**0.012**
*Intervention* (ref: Control)						
Patients	0.15	−0.66, 0.97	0.714	−0.25	−1.16, 0.66	0.588
Psychiatrists	0.02	−0.61, 0.64	0.958	0.11	−0.60, 0.83	0.754
Time* Intervention (ref: Control)						
Patients	−0.02	−0.08, 0.03	0.377	0.08	0.004, 0.15	**0.039**
Psychiatrists	−0.03	−0.07, 0.02	0.208	0.05	-0.003, 0.11	0.062
*Age*	−0.05	−0.07, −0.02	**0.001**	–	–	–
*Marital status: Single/widowed/separated* (ref: In a couple)	–	–	–	−0.70	−1.29, −0.11	**0.020**
Intercept	15.68	13.99, 17.38	<0.001	16.67	16.04, 17.29	<0.001

Health-related quality of life: Physical and Mental.

QoL, quality of life; SF-12, Short Form-12. Bold values are statistically significant.

## 4 Discussion

The main objective of this RCT was to evaluate long-term effectiveness of a multicomponent strategy to improve medication adherence in adult patients diagnosed with a depressive disorder. No effect of PsI or PtI on patients’ adherence to antidepressant treatment was found. However, PsI and PtI decreased the severity of depressive symptoms. Moreover, an improvement in the mental QoL of patients with depressive disorders was observed in PtI intervention group, even over the long term. Patients in PsI also exhibited a similar trend.

Previous research has demonstrated the effectiveness of these types of interventions improving adherence to antidepressant treatments among patients with depressive disorders at 3 and 6-months after intervention (24). However, in the present study, no significant differences were observed in therapeutic adherence between patients who received the intervention, patients whose healthcare professionals underwent intervention or those who received usual care. This lack of differences could potentially be attributed to the fact that most patients included in the study were adherent to antidepressant treatment at baseline, approximately 75% of the patients, which is not consistent with the majority of previous literature (11–16). This leads one to think that, on the one hand, these groups could have some different characteristics that influence the results. On the other hand, the high initial adherence rates could be creating a ceiling effect on the potential effectiveness of the intervention. Moreover, the implementation of two separate interventions, one targeting the professionals and the other targeting the patients might have mitigated the overall impact. Therefore, the authors believe that future studies that combine both interventions are necessary (24). Additionally, it should be noted that the sample size may not have reached the theoretical size needed to detect statistically significant differences.

However, a decrease was found in the severity of depressive symptoms in both intervention groups (patients and psychiatrists) throughout the follow-up periods. While current evidence does not definitively establish whether SDM interventions improve clinical outcomes, such as depression (43), the findings here provide support for the notion that training healthcare professionals in SDM, particularly when combined with enhanced motivational interviewing skills (44), can have a positive impact on the severity levels of patients with depression. This suggests that implementing SDM, along with improved motivational interviewing techniques, may be beneficial in managing depression in these patients. Further research is warranted to better understand the specific mechanisms and long-term effects of these interventions on depression outcomes. These results are consistent with that observed in the literature. It has been shown that multicomponent strategies applied in patients improve symptoms of depression (45). In this respect, it seems that components of demonstrated effectiveness, such as psychoeducation (46) and behavioral activation based intervention (47, 48) can contribute to the reduction of depressive symptoms. This identifies the need to improve the training and strengthening of patients in self-motivation, self-control and monitoring of their disease.

In the present study, surprisingly, it has been observed that comorbid anxiety increased after the intervention in the PtI and PsI groups. These findings may be conditioned by the nature of the interventions, which although focused primarily on emphasizing antidepressant adherence as the central component of treatment, also highlighted the importance of adequately informing patients about the need to reduce benzodiazepine consumption. Patients were provided with detailed instructions on withdrawal, including information about potential withdrawal effects and solutions to address them. Consequently, future studies incorporating these aspects into their interventions are necessary to validate these results.

Finally, an improvement in the mental QoL of patients was observed in PtI intervention group. This effect on QoL has been observed in other interventions carried out in chronic diseases (49, 50). A trend toward improvement was also found especially in the PsI group. In this case, the intervention consisted of an update of evidence-based clinical knowledge in PCC, including SDM (51). In the case of depression, those strategies that consider the patient’s beliefs regarding depression are essential for the success of the therapy (52), which could be related to these results.

As mentioned above, the main limitation of this study is that most of the participants with depressive disorders included in this study present high adherence to their antidepressant treatment regimen according to the Sidorkiewicz scale, which is contrary to that found in the literature. This suggests that the sample used here behaves differently to how the general population would. The potential for a selection bias could arise from the involvement of psychiatrists in the patient selection process. This study is not double-blind which allows for the presence of information biases such as the Hawthorne effect (patients change their behavior in response to their awareness of being watched), social desirability bias (patients report in overdoing positive behaviors or underestimating undesirable ones) and performance bias (doctors modify their behavior). On the other hand, as has been mentioned, adherence is a multifactorial phenomenon (53, 54), and as such it should ideally be evaluated from different points of view. For this reason, potential shortcomings associated with relying solely on a questionnaire for assessing adherence are compounded. Using a single subjective measurement method, although it is estimated that the probability of obtaining overestimated adherence results is reduced using the Sidorkiewicz scale, may be insufficient. Therefore, these findings should be confirmed through objective measures of adherence. Another relevant limitation is the limited sample size to analyze interaction effects. At the time the study was carried out, there was a pandemic situation due to COVID, which made recruiting patients and professionals extremely difficult. The results to be drawn are therefore limited by the low statistical power for the analyses.

In conclusion, interventions for patients and psychiatrists decrease the severity of depressive symptoms. Moreover, these interventions improve mental QoL in patients with depressive disorders, even over the long term. However, no effect on antidepressant adherence was found. Nevertheless, these results should be confirmed in future research studies.

## Data availability statement

The raw data supporting the conclusions of this article will be made available by the authors, without undue reservation.

## Ethics statement

The studies involving humans were approved by Comité ético de Investigación Clínica del Hospital Universitario de Canarias Hospital Universitario de Canarias. The studies were conducted in accordance with the local legislation and institutional requirements. The participants provided their written informed consent to participate in this study.

## Author contributions

TP-S: Writing – original draft, Methodology, Investigation, Formal analysis, Data curation, Conceptualization. HG-P: Writing – original draft, Formal analysis. BG: Writing – original draft, Methodology, Investigation, Conceptualization. PS-P: Writing – review & editing, Formal analysis, Conceptualization. FA: Writing – review & editing, Formal analysis. CV-N: Writing – original draft, Formal analysis. IH-N: Writing – review & editing, Formal analysis. CR: Writing – review & editing, Formal analysis. MT-M: Writing – review & editing, Writing – original draft, Validation, Supervision, Resources, Project administration, Methodology, Investigation, Conceptualization.

## References

[1] Vos T Lim SS Abbafati C Abbas KM Abbasi M Abbasifard M. Global burden of 369 diseases and injuries in 204 countries and territories, 1990–2019: a systematic analysis for the global burden of disease study 2019. Lancet. (2020) 396:1204–22. doi: 10.1016/S0140-6736(20)30925-9, PMID: 33069326 PMC7567026

[2] Moreno-Agostino D Wu Y-T Daskalopoulou C Hasan MT Huisman M Prina M. Global trends in the prevalence and incidence of depression:a systematic review and meta-analysis. J Affect Disord. (2021) 281:235–43. doi: 10.1016/j.jad.2020.12.035, PMID: 33338841

[3] Santomauro DF Mantilla Herrera AM Shadid J Zheng P Ashbaugh C Pigott DM. Global prevalence and burden of depressive and anxiety disorders in 204 countries and territories in 2020 due to the COVID-19 pandemic. Lancet. (2021) 398:1700–12. doi: 10.1016/S0140-6736(21)02143-7, PMID: 34634250 PMC8500697

[4] Lawrence D Hancock KJ Kisely S. The gap in life expectancy from preventable physical illness in psychiatric patients in Western Australia: retrospective analysis of population based registers. BMJ. (2013) 346:f2539–9. doi: 10.1136/bmj.f2539, PMID: 23694688 PMC3660620

[5] Favril L Yu R Uyar A Sharpe M Fazel S. Risk factors for suicide in adults: systematic review and meta-analysis of psychological autopsy studies. Evid Based Ment Health. (2022) 25:148–55. doi: 10.1136/ebmental-2022-300549, PMID: 36162975 PMC9685708

[6] World Health Organization (2022). Mental health at work. Available at: https://www.who.int/news-room/fact-sheets/detail/mental-health-at-work (accessed May 7, 2023).

[7] Noguchi T Saito M Aida J Cable N Tsuji T Koyama S. Association between social isolation and depression onset among older adults: a cross-national longitudinal study in England and Japan. BMJ Open. (2021) 11:e045834. doi: 10.1136/bmjopen-2020-045834, PMID: 33737442 PMC7978252

[8] The Lancet. The health crisis of mental health stigma. Lancet. (2016) 387:1027. doi: 10.1016/S0140-6736(16)00687-5, PMID: 27025171

[9] Hunt GE Malhi GS Lai HMX Cleary M. Prevalence of comorbid substance use in major depressive disorder in community and clinical settings, 1990–2019: systematic review and meta-analysis. J Affect Disord. (2020) 266:288–04. doi: 10.1016/j.jad.2020.01.141, PMID: 32056890

[10] National Institute for Health and Care Excellence (2022). Depression in adults: treatment and management: NICE guideline. Available at: www.nice.org.uk/guidance/ng222. (Accessed August 17, 2023). 35977056

[11] Holvast F Oude Voshaar RC Wouters H Hek K Schellevis F Burger H. Non-adherence to antidepressants among older patients with depression: a longitudinal cohort study in primary care. Fam Pract. (2019) 36:12–20. doi: 10.1093/fampra/cmy106, PMID: 30395196

[12] Sansone RA Sansone LA. Antidepressant adherence: are patients taking their medications? Innov Clin Neurosci. (2012) 9:41–6. PMID: 22808448 PMC3398686

[13] Ten Doesschate MC Bockting CLH Schene AH. Adherence to continuation and maintenance antidepressant use in recurrent depression. J Affect Disord. (2009) 115:167–70. doi: 10.1016/j.jad.2008.07.011, PMID: 18760488

[14] Baeza-Velasco C Olié E Béziat S Guillaume S Courtet P. Determinants of suboptimal medication adherence in patients with a major depressive episode. Depress Anxiety. (2019) 36:244–51. doi: 10.1002/da.22852, PMID: 30328659

[15] Párraga Martínez I López-Torres Hidalgo J del Campo del Campo JM Villena Ferrer A Morena Rayo S Escobar Rabadán F. Seguimiento de la adherencia al tratamiento antidepresivo en pacientes que inician su consumo. Aten Primaria. (2014) 46:357–66. doi: 10.1016/j.aprim.2013.11.003, PMID: 24704196 PMC6983598

[16] Pedrosa-Naudín MA Gutiérrez-Abejón E Herrera-Gómez F Fernández-Lázaro D Álvarez FJ. Non-adherence to antidepressant treatment and related factors in a region of Spain: a population-based registry study. Pharmaceutics. (2022) 14:2696. doi: 10.3390/pharmaceutics14122696, PMID: 36559190 PMC9782667

[17] Ho SC Chong HY Chaiyakunapruk N Tangiisuran B Jacob SA. Clinical and economic impact of non-adherence to antidepressants in major depressive disorder: a systematic review. J Affect Disord. (2016) 193:1–10. doi: 10.1016/j.jad.2015.12.029, PMID: 26748881

[18] Cantrell CR Eaddy MT Shah MB Regan TS Sokol MC. Methods for evaluating patient adherence to antidepressant therapy: a real-world comparison of adherence and economic outcomes. Med Care. (2006) 44:300–3. doi: 10.1097/01.mlr.0000204287.82701.9b, PMID: 16565629

[19] Henein F Prabhakar D Peterson EL Williams LK Ahmedani BK. A prospective study of antidepressant adherence and suicidal ideation among adults. Prim Care Companion CNS Disord. (2016). doi: 10.4088/PCC.16l01935, PMID: 27907275 PMC5997580

[20] Wade AG Häring J. A review of the costs associated with depression and treatment noncompliance: the potential benefits of online support. Int Clin Psychopharmacol. (2010) 25:288–96. doi: 10.1097/YIC.0b013e328339fbcf, PMID: 20715299

[21] Mert DG Turgut NH Kelleci M Semiz M. Perspectives on reasons of medication nonadherence in psychiatric patients. Patient Prefer Adherence. (2015) 9:87–93. doi: 10.2147/PPA.S75013 25609930 PMC4298301

[22] Murata A Kanbayashi T Shimizu T Miura M. Risk factors for drug nonadherence in antidepressant-treated patients and implications of pharmacist adherence instructions for adherence improvement. Patient Prefer Adherence. (2012) 6:863–9. doi: 10.2147/PPA.S36295, PMID: 23271895 PMC3526883

[23] Sawada N Uchida H Suzuki T Watanabe K Kikuchi T Handa T. Persistence and compliance to antidepressant treatment in patients with depression: a chart review. BMC Psychiatry. (2009) 9:38. doi: 10.1186/1471-244X-9-38, PMID: 19531229 PMC2702377

[24] González De León B Del Pino-Sedeño T Serrano-Pérez P Rodríguez Álvarez C Bejarano-Quisoboni D Trujillo-Martín MM. Effectiveness of interventions to improve medication adherence in adults with depressive disorders: a meta-analysis. BMC Psychiatry. (2022) 22:487. doi: 10.1186/s12888-022-04120-w 35858887 PMC9301839

[25] Moriarty AS Coventry PA Hudson JL Cook N Fenton OJ Bower P. The role of relapse prevention for depression in collaborative care: a systematic review. J Affect Disord. (2020) 265:618–44. doi: 10.1016/j.jad.2019.11.105, PMID: 31791677

[26] Sighinolfi C Nespeca C Menchetti M Levantesi P Belvederi Murri M Berardi D. Collaborative care for depression in European countries: a systematic review and meta-analysis. J Psychosom Res. (2014) 77:247–63. doi: 10.1016/j.jpsychores.2014.08.006, PMID: 25201482

[27] del Pino-Sedeño T Peñate W de Las Cuevas C Valcarcel-Nazco C Fumero A Serrano-Pérez PG. Effectiveness and cost-effectiveness of a multicomponent intervention to improve medication adherence in people with depressive disorders - MAPDep: a study protocol for a cluster randomized controlled trial. Patient Prefer Adherence. (2019) 13:309–19. doi: 10.2147/PPA.S172963, PMID: 30863020 PMC6391125

[28] González De León B León Salas B Del Pino-Sedeño T Rodríguez-Álvarez C Bejarano-Quisoboni D Trujillo-Martín MM. Aplicaciones móviles para mejorar la adherencia a la medicación: revisión y análisis de calidad. Aten Primaria. (2021) 53:102095. doi: 10.1016/j.aprim.2021.102095, PMID: 34139398 PMC8213909

[29] de las Cuevas C Peñate W Manuel García de Cecilia J de Leon J. Predictive validity of the Sidorkiewicz instrument in Spanish: assessing individual drug adherence in psychiatric patients. Int J Clin Health Psychol. (2018) 18:133–42. doi: 10.1016/j.ijchp.2017.11.003, PMID: 30487918 PMC6225059

[30] Herrero MJ Blanch J Peri JM De Pablo J Pintor L Bulbena A. A validation study of the hospital anxiety and depression scale (HADS) in a Spanish population. Gen Hosp Psychiatry. (2003) 25:277–83. doi: 10.1016/S0163-8343(03)00043-4, PMID: 12850660

[31] Sanz J Perdigón AL Vázquez C. Adaptación española del Inventario para la Depresión de Beck-II (BDI-II). Clínica Salud. (2003) 14:249–80.

[32] Gandek B Ware JE Aaronson NK Apolone G Bjorner JB Brazier JE. Cross-validation of item selection and scoring for the SF-12 health survey in nine countries. J Clin Epidemiol. (1998) 51:1171–8. doi: 10.1016/S0895-4356(98)00109-7, PMID: 9817135

[33] Ware JE Kosinski M Keller SD. A 12-item short-form health survey: construction of scales and preliminary tests of reliability and validity. Med Care. (1996) 34:220–33. doi: 10.1097/00005650-199603000-00003, PMID: 8628042

[34] Button KS Kounali D Thomas L Wiles NJ Peters TJ Welton NJ. Minimal clinically important difference on the Beck depression inventory - II according to the patient’s perspective. Psychol Med. (2015) 45:3269–79. doi: 10.1017/S0033291715001270, PMID: 26165748 PMC4611356

[35] Resnick B Nahm ES. Reliability and validity testing of the revised 12-item short-form health survey in older adults. J Nurs Meas. (2001) 9:151–61. doi: 10.1891/1061-3749.9.2.151, PMID: 11696939

[36] Su S-W Wang D. The reliability and validity of short Form-12 health survey version 2 for Chinese older adults. Iran J Public Health. (2019) 48:1014–24. PMID: 31341842 PMC6635330

[37] Robles García R Salazar Alvarado V Páez Agraz F Ramírez Barreto F. Assessment of drug attitudes in patients with schizophrenia: psychometric properties of the DAI Spanish version. Actas Esp Psiquiatr. (2004) 32:138–42. PMID: 15168263

[38] De Las Cuevas C Rivero-Santana A Perestelo-Perez L Gonzalez-Lorenzo M Perez-Ramos J Sanz EJ. Adaptation and validation study of THE beliefs about medicines questionnaire in psychiatric outpatients in a community mental health setting: adaptation and validation of the BMQ. Hum Psychopharmacol Clin Exp. (2011) 26:140–6. doi: 10.1002/hup.1185 21455972

[39] Tomás-Sábado J Montes-Hidalgo J. Versión española de la Escala multidimensional de locus de control de la salud en estudiantes de enfermería. Enferm Clínica. (2016) 26:181–7. doi: 10.1016/j.enfcli.2015.12.005 26922046

[40] De Las Cuevas C Peñate W. Validity of the control preferences scale in patients with emotional disorders. Patient Prefer Adherence. (2016) 10:2351–6. doi: 10.2147/PPA.S122377 27895470 PMC5118017

[41] De Las Cuevas C Peñate W Betancort M De Rivera L. Psychological reactance in psychiatric patients: examining the dimensionality and correlates of the Hong psychological reactance scale in a large clinical sample. Personal Individ Differ. (2014) 70:85–91. doi: 10.1016/j.paid.2014.06.027

[42] StataCorp (2021). Stata Statistical Software.

[43] Aoki Y Yaju Y Utsumi T Sanyaolu L Storm M Takaesu Y. Shared decision-making interventions for people with mental health conditions. Cochrane Database Syst Rev. (2022) 11:CD007297. doi: 10.1002/14651858.CD007297.pub3 36367232 PMC9650912

[44] Baricchi M Vellone E Caruso R Arrigoni C Dellafiore F Ghizzardi G. Technology-delivered motivational interviewing to improve health outcomes in patients with chronic conditions: a systematic review of the literature. Eur J Cardiovasc Nurs. (2023) 22:227–35. doi: 10.1093/eurjcn/zvac071, PMID: 35943381

[45] Williams JW Gerrity M Holsinger T Dobscha S Gaynes B Dietrich A. Systematic review of multifaceted interventions to improve depression care. Gen Hosp Psychiatry. (2007) 29:91–16. doi: 10.1016/j.genhosppsych.2006.12.003, PMID: 17336659

[46] Katsuki F Watanabe N Yamada A Hasegawa T. Effectiveness of family psychoeducation for major depressive disorder: systematic review and meta-analysis. BJPsych Open. (2022) 8:e148. doi: 10.1192/bjo.2022.543, PMID: 35915980 PMC9380172

[47] Orgeta V Brede J Livingston G. Behavioural activation for depression in older people: systematic review and meta-analysis. Br J Psychiatry. (2017) 211:274–9. doi: 10.1192/bjp.bp.117.205021, PMID: 28982660

[48] Tindall L Mikocka-Walus A McMillan D Wright B Hewitt C Gascoyne S. Is behavioural activation effective in the treatment of depression in young people? A systematic review and meta-analysis. Psychol Psychother Theory Res Pract. (2017) 90:770–96. doi: 10.1111/papt.12121, PMID: 28299896 PMC5697579

[49] Waddington F Amerikanou M Brett J Watson E Abbots V Dawson P. A systematic review to explore the effectiveness of physical health and psychosocial interventions on anxiety, depression and quality of life in people living with blood cancer. J Psychosoc Oncol. (2024) 42:113–147. doi: 10.1080/07347332.2023.2228309 37401811

[50] Wojeck RK Arcoleo K Hathaway EC Somers TJ. Nurse-led interventions in systemic autoimmune rheumatic diseases: a systematic review. BMC Nurs. (2023) 22:232. doi: 10.1186/s12912-023-01393-8 37400809 PMC10318744

[51] Xu RH Zhou L Wang D. The relationship between decisional regret and well-being in patients with and without depressive disorders: mediating role of shared decision-making. Front Psych. (2021) 12:657224. doi: 10.3389/fpsyt.2021.657224, PMID: 34220572 PMC8242166

[52] Acosta F Rodríguez L Cabrera B. Creencias sobre la depresión y sus tratamientos: variables asociadas e influencia de las creencias en la adherencia. Rev Psiquiatr Salud Ment. (2013) 6:86–92. doi: 10.1016/j.rpsm.2012.08.001, PMID: 23084794

[53] Brown MT Bussell J Dutta S Davis K Strong S Mathew S. Medication adherence: truth and consequences. Am J Med Sci. (2016) 351:387–99. doi: 10.1016/j.amjms.2016.01.010 27079345

[54] Tan JP Cheng KKF Siah RC. A systematic review and meta-analysis on the effectiveness of education on medication adherence for patients with hypertension, hyperlipidaemia and diabetes. J Adv Nurs. (2019) 75:2478–94. doi: 10.1111/jan.14025, PMID: 30993749

